# Five Years On and Still in Motion: A Retrospective Analysis of the Five-Year Survivorship of Dual Mobility Total Hip Arthroplasty Performed for Diverse Indications

**DOI:** 10.7759/cureus.97614

**Published:** 2025-11-23

**Authors:** Suvank Rout, Sanjay Chauhan, Souvagya Rout

**Affiliations:** 1 Orthopaedics, Topiwala National Medical College and B.Y.L. Nair Charitable Hospital, Mumbai, IND; 2 Trauma and Orthopaedics, Colchester General Hospital, Colchester, GBR; 3 Trauma and Orthopaedics, King Edward Memorial Hospital and Seth Gordhandas Sunderdas Medical College, Mumbai, IND; 4 Trauma and Orthopaedics, First Faculty of Medicine, Charles University, Prague, CZE

**Keywords:** dual-mobility hip arthroplasty, dual mobility total hip arthroplasty, functional outcome of total hip aethroplasty, hip joint replacement, hybrid total hip arthroplasty, total joint arthroplasties

## Abstract

Background

One of the most common causes of total hip Arthroplasty (THA) failures is instability and dislocation of the implant. Dual Mobility (DM) Liners were developed to help mitigate this risk.

Aim of the study

This study aims to study the functional at subjective results of Dual mobility THA performed for various purposes at a tertiary centre hospital in Mumbai, with a minimum post-operative period of five years.

Methodology

The study was conducted as a retrospective study in a tertiary health care hospital in a metropolitan city over a period of one year. Patients were examined clinically, and questions were asked to calculate the Harris hip score and Oxford hip score. Fresh plain radiographs were taken and evaluated. The mid-term post-operative results were analysed. We recruited 60 participants with a mean age of 65.77 years with SD 10.13, and the age range was between 35-88; and 53.3% of them (n = 28) were female.

Results

The range of post-operative period at the time of participant recruitment was 5-8 years, with a mean of 6.15 ± 0.77. Out of the total 60 patients, we observed complications in only four patients, which included persistent thigh pain in the post-op period (n=2), heterotrophic calcification in the post-op period (n=1), and revision THR at six years post-operatively (n=1). We also observed that only one participant developed aseptic loosening of the hip postoperatively.

The clinical outcomes and observed the mean Harris’ hip score to be 87.38 ± 4.41 in our population, and the mean Oxford hip score in our population was 37.35 ± 4.60.  The observed mean score for Moore’s criteria for osteointegration was 3.10 ± 0.47. Most of the participants reported their post-operative condition to be excellent (60%), while 22% reported it to be good and 10% reported it to be fair.

Implications

A statistically significant association between the clinical outcomes and the subjective outcomes, indicating that participants who had better Harris’ Hip scores and Oxford hip scores were more likely to subjectively feel their post-operative condition to be better. A statistically significant association between the radiological outcomes and the subjective outcomes, indicating that participants who had better Moore’s criteria for osteointegration were more likely to subjectively feel their post-operative condition to be better. No statistically significant association between age and the Harris hip score and Oxford hip scores, indicating that the clinical outcomes were similar across all age groups. However, we observed a statistically significant difference between age and Moore’s criteria for osteointegration, indicating that the radiological outcomes were worse with advancing age.

## Introduction

Total hip arthroplasty (THA) is a surgical procedure in which the hip joint is replaced completely by a prosthetic implant, referred to as a hip prosthesis. THA represents about 1.5 million surgeries performed worldwide each year [1]. THA is known to last at least 25 years in 58% of all total hip replacements (THR), suggesting it is one of the most successful surgeries [1]. The aims of performing a THA include pain relief and improvement in hip function. This modality of treatment is usually considered only after other therapies, such as physical therapy and pain medications, have failed to provide pain relief or relief from other symptoms. 

The earliest recorded attempts at hip replacement were carried out by Themistocles Gluck in 1891 using ivory to replace the femoral head, attaching it with nickel-plated screws, plaster of Paris, and glue [2,3]. Dr Austin T. Moore in Columbia, South Carolina 1940 performed the first metallic hip replacement surgery, where he used a fixed head proximal femoral implant made of cobalt-chrome alloy vitallium [2,3]. 

THA is most commonly indicated in the treatment of joint failure caused by osteoarthritis. The incidence of THAs varies in developed countries between 30-290 procedures per 100,000 population per year [4-5]. Even though associated with a good success rate, the two main concerns still persist with THA are dislocation and wear. During THA, the extracapsular ligaments (the iliofemoral, ischiofemoral, and pubofemoral ligaments), which are responsible for preventing dislocation of the native hip, are excised, which might result in this phenomenon. Dislocations of the prosthesis are noted to mostly occur in the first three months post insertion, as the soft tissues surrounding the joint are relaxed and there is incomplete scar formation [6]. Studies note that it takes about 8-12 weeks for soft tissues to heal that have been injured or cut during surgery. If a large femoral head diameter is used and a small amount of soft tissue is cut, this tends to reduce the risk of dislocations [6]. Malposition of the implant prosthesis or dysfunction of muscles results in dislocation of the prosthesis between three months to five years post-surgery [6]. After five year duration, the risk factors for dislocation include female gender, younger age at primary hip arthroplasty, previous subluxation without complete dislocation, previous trauma, substantial weight loss, recent onset or progression of dementia or a neurological disorder, malposition of the cup, wear of the liner (particularly when it causes movement of the head of more than 2 mm within the cup compared to its original position) and prosthesis loosening with migration [6].

Instability has been reported as the leading complication in the first year after primary and revision THA according to the England and Wales National Joint Registry report [7]. Early clinical reports, mainly from France, have shown that dual mobility (DM) THR can reduce dislocation rates in primary THA [8]. This is supported by more recent data from newer DM hips [9]. It seems increasingly clear that using DM is likely to offer advantages to patients. This would include particularly those patients who are at higher risk of instability, either due to lack of muscle control, neuromuscular issues, soft-tissue problems, or spino-pelvic balance problems. To address these problems of dislocation and instability, Professor Gilles Bousquet and André Rambert invented the DM concept and patented it in 1975 [10]. The DM hip concept has resulted in the improvement in range of motion and seems to significantly reduce the risk of dislocations and instability; the DM implants also have been reported to exhibit some specific complications secondary to their specific design, with the presence of a third joint. For instance, intra-prosthetic dislocation has appeared as a new issue.

Intra-prosthetic dislocation occurs due to retentive failure of the polyethylene (PE) liner on the femoral head is a complication observed exclusively with the DM implant and involves the articulation failure between the femoral head and the PE liner [11]. In an intra-prosthetic dislocation, the inner prosthetic femoral head disengages from the outer PE bearing due to abnormal PE wear. Also, wear of the implant is still a concern even with a DM type, as this can result in osteolysis secondary to the release of particles from the implant. 

The presence of two articulations in the design of the implant results in an increase in the range of motion (ROM) of the implant before impingement can occur. In the primary articulation, the femoral head is “engaged” within the PE liner, maintaining mobility and exhibiting the typical mechanical behaviour of a hard-on-soft bearing, as seen in conventional THA. When contact occurs between the femoral neck and the rim of the PE liner, a secondary articulation is activated. This articulation involves movement between the outer surface of the PE liner and the inner surface of the metallic acetabular shell. As the PE liner rotates within the shell, the effective ROM is further increased until impingement occurs between the femoral neck and the rim of the shell. In this manner, the head-liner complex functions analogously to a large femoral head, increasing the head-neck ratio and, consequently, the jump distance before dislocation. Despite these theoretical advantages in stability and ROM, DM THA may still be associated with long-term complications, particularly related to instability or dislocation.

Hence, we conducted this study to evaluate the midterm results of DM THA done for various purposes. We took into account both clinical outcomes and radiological outcomes. The clinical outcomes included were signs and symptoms such as pain, hip function, absence of deformity and range of motion; and the incidence of complications such as hip dislocations, intra-prosthetic dislocation, aseptic loosening, osteolysis and stress shielding. The radiographic findings of the DM system were also assessed using various hip scores in serial radiographic assessments.

## Materials and methods

Study design

We performed a retrospective study at a single tertiary health care hospital in a metropolitan city and conducted it over a period of one year. Patients who underwent primary or revision Dual mobility total hip arthroplasty were assessed in the outpatient clinics during their follow-up. They were examined clinically, and questions were asked to calculate the Harris hip score and Oxford hip score. Fresh plain radiographs were taken and evaluated. Various data would have been collected from patients coming to the outpatient clinic for regular follow-up following dual mobility total hip arthroplasty over a period of time and stored in the unit database. These data include scores of clinical parameters like the Harris hip score, the Oxford hip score and serial radiographs. The mid-term post-operative results were analysed.

Surgical technique and implants

All the procedures were performed using a standardised posterior approach, with appropriate repair of soft tissue and capsule. The acetabular component used in all cases was the triology acetabular system (Zimmer Biomet), which was coupled with its highly cross-linked polyethylene Dual Mobility liner. Acetabular components were press-fitted, and supplemental screw fixation was used as per intra-operative stability requirements. Femoral stems used were either cementless or cemented, as per the patient's bone quality and surgeon preference. All the patients received standardised perioperative antibiotic prophylaxis and thromboembolism prophylaxis.

Inclusion criteria

The Inclusion criteria were patients with an age of >18 years. Both male and female patients were included in this study. The ethnicity of the patients was that of Asian-Indian Origin. Patients who underwent dual mobility total hip arthroplasty for any hip pathology.

Exclusion criteria

Patients not willing to provide written informed consent were not included in this study. Patients whose five-year follow-up is not available were excluded. A total of 169 hips in 157 patients were identified and included in the final analysis.

Assessment of outcomes

The assessment on outcomes was based on three criteria, which were: Clinical outcomes, radiological outcomes and incidence of complications.

Clinical outcomes were assessed on the basis of A. Harris Hip Score (HHS), B. Oxford Hip Score (OHS). Radiological outcomes were assessed by Moore's Criteria for osteointegration in THR. Incidence of complications took into account A. Hip dislocations, B. Intra-prosthetic dislocations, C. Aseptic loosening, D. Osteolysis and E. Stress shielding. Aseptic loosening is often diagnosed with patient reporting new pain or an increase in pain on weight bearing or minimal range of motion and > 2mm radiolucent lines around the femoral or acetabular components, a shift in the implant's position, or osteolysis (bone loss) in the femur or pelvis.

Harris Hip Score

The score was developed for the assessment of the results of hip surgery, and is intended to evaluate various hip disabilities and methods of treatment. The domains covered are pain, function, absence of deformity (fixed flexion contracture, abduction, internal rotation, and limb length discrepancy) and range of motion. The score has a maximum of 100 points (best possible outcome) covering pain (1 item, 0-44 points), function (7 items, 0-47 points), absence of deformity (1 item, 4 points) and range of motion (2 items, 5 points) [12].

Oxford Hip Score

The Oxford Hip Score (OHS) is a patient-reported outcome measure (PROM) designed to assess pain and function in patients with hip pathology, especially those undergoing total hip replacement. It assesses two domains, namely pain and function of the hip. These two domains consist of six items or questions in each. In the scoring system, items are scored from 0 to 4, where 0 represents the worst outcome and 4 represents the best [13].

Moore's Criteria

Moore’s criteria for osteointegration are defined by five radiographic signs for detecting acetabular osseointegration: A) Absence of radiolucent lines; B) Presence of a supero-lateral buttress; C) Medial stress shielding; D) Radial trabeculae; and E) An infero-medial buttress [13]. When three or more signs were present, the positive predictive value of the radiographic test was 96.9%, the sensitivity was 89.6%, and the specificity was 76.9%. The five signs of acetabular osseointegration reliably predicted osseointegration, especially when used in combination [14].

Data analysis

Categorical and nominal data are expressed as numbers and percentages. Quantitative data have been represented as mean ± standard deviation. Independent-sample t-tests were used for numerical data. Paired t-tests were used to compare measures before and after surgery. The chi-square test was used for categorical data. Statistical significance was set at P <0.05 for two-sided probabilities. Implant survivorship was analysed using the Kaplan-Meier method, with 95% confidence intervals. All analysis was carried out using Microsoft Excel.

## Results

Demographic distribution

It was found that out of the total 60 study participants, most of them, 32 (53.3%), belonged to the 61-70 years of age (Table 1). Twelve of them (20%) belonged to 71-80 years of age, four participants (6.7%) belonged to >80 years of age and seven participants (11.7%) belonged to 51-70 years, four participants (6.7%) belonged to 41-50 years of age and only one (1.7%) study participant belonged to (1.7%) study participant belonged to <40 years of age. The mean age of study participants was 65.77 years, with a standard deviation (SD) of 10.13, and the range was between 35-88. It was also found that out of the total 60 patients, 28 (46.7%) were male and 32 (53.3%) were female (Table 2).

**Table 1:** Demographic characteristics of the study cohort (N=60) Data presented as number of subjects (Percentage) unless otherwise specified.

Age (Years)	Number of patients (Percentage of study cohort)
≤40	1 (1.7)
41-50	4 (6.7)
51-60	7 (11.7)
61-70	32 (53.3)
71-80	12 (20.0)
>80	4 (6.7)

**Table 2:** Gender distribution of study subjects (N=60) *Note: Chi-square goodness-of-fit test showed no significant deviation from equal distribution (χ² = 0.267, p = 0.605)*.

Gender	Number of patients (Percentage of study cohort)
Male	28(46.7%)
Female	32 (53.3%)
Chi-square test	0.267
p- score	0.605

Out of the total 60 patients, we observed that 12 (20%) were five years post-operative, 1 (1.7%) was 5.5 years post-operative, 25 (41.7%) were six years post-operative, 21 (35%) were seven years post-operative, and only 1 (1.7%) was eight years post-operative (Table 3). The mean postoperative duration was 6.15 years with SD 0.77, and the range was between 5-8 years.

**Table 3:** Study population stratified according to post-operative follow-up time (N=60)

Duration post-op (years)	Number	Percentages
5	12	20.00%
5.5	1	1.70%
6	25	41.70%
7	21	35.00%
8	1	1.70%

Implant survivorship and complications

In our study, it was found that out of the total 60 patients, only one (1.7%) had aseptic loosening post-op, while 59 (98.3%) did not (Table 4). We also found that out of the total 60 patients, 56 (93.3%) had no complications, while four (6.7%) had some complications (Table 5). Out of a total of four patients, one (1.7%) had heterotrophic calcification in the post op period, two (3.3%) had persistent thigh pain in the post-op period, and one (1.7%) had undergone revision THR at six years post-operatively (Table 5).

**Table 4:** Study subjects grouped according to aseptic loosening (N=60).

Aseptic loosening	Number	Percentage
Present	1	1.7%
No aseptic Loosening	59	98.3%

**Table 5:** Distribution of study subjects according to the post-operative complications (N=60)

Complications	Number	Percentages (%)
No Complication	56	93.3
Heterotrophic Calcification	1	1.7
Persistent Thigh Pain	2	3.3
Revision THR at 6 years	1	1.7
Total	60	100

**Figure 1:**
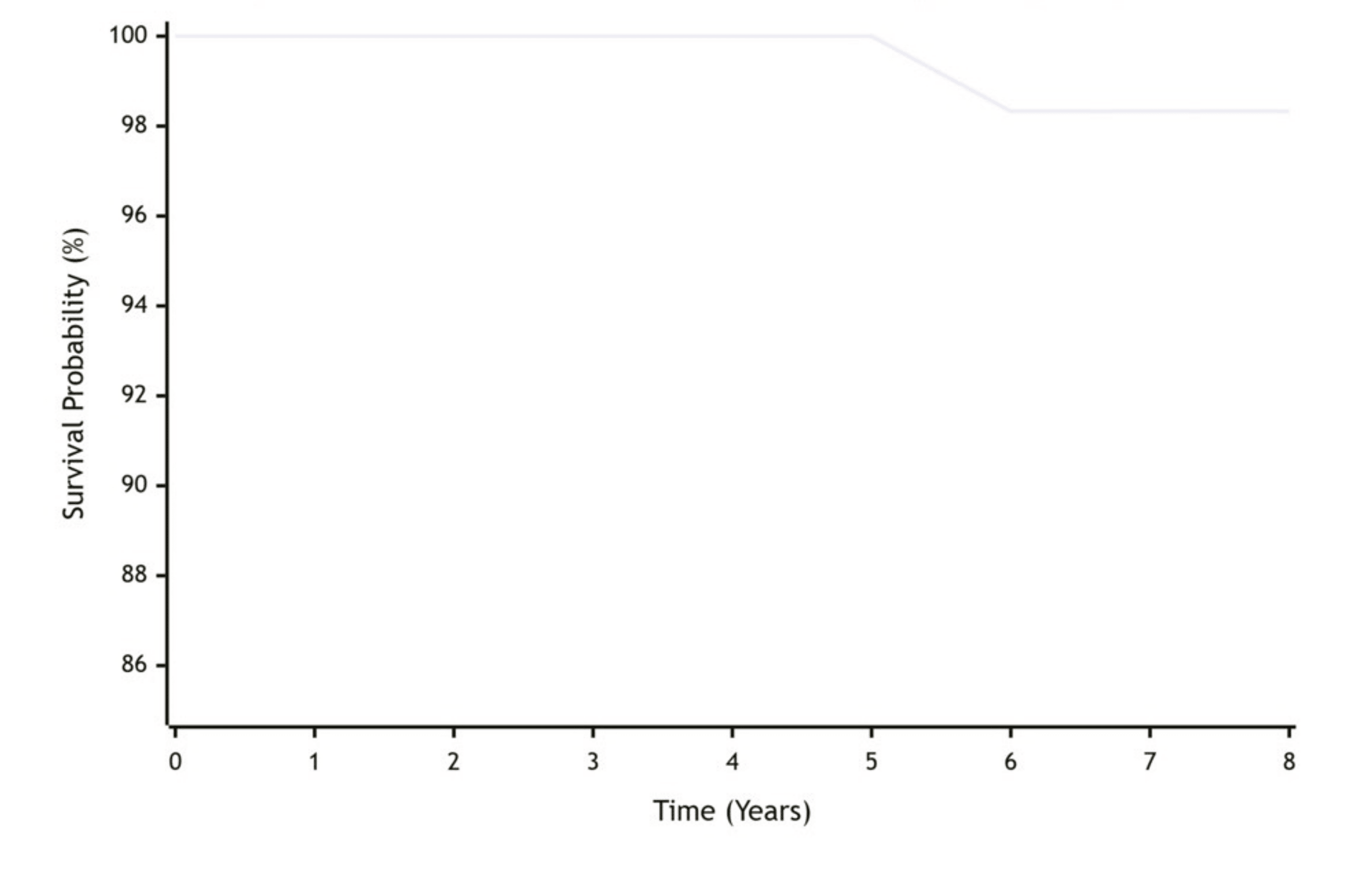
Kaplan-Meier survival curve for dual mobility total hip arthroplasty (THA)

We noted that the all-cause survivorship of the dual-mobility implant was 98.33% (95% Confidence Interval: 94.1-99.1) at eight years, which is determined by the Kaplan-Meier analysis (Figure 1). There was one revision in our cohort of patients at the six-year postoperative duration. After the single revision at six years, the probability remains stable at 98.33% through eight years of follow-up. The number of patients at risk gradually decreases over time due to patients not reaching longer follow-up milestones, which is normal in longitudinal studies.

Subjective and functional outcomes 

We also observed that out of the total 60 patients, 36 (60%) had excellent subjective outcomes, 11 (18.3%) had fair outcomes, and the remaining 13 (21.7%) had good outcomes post-operatively (Table 6).

**Table 6:** Study population stratified by subjective outcome (N=60)

Subjective outcomes	Number	Percentage (%)
Excellent	36	60.0
Fair	11	18.3
Good	13	21.7

We compared the subjective outcomes in our participants with their Harris hip scores, Oxford hip score and Moore’s criteria for osteointegration. We found that out of the total 60 patients, who were having excellent outcomes, had a mean Harris Hip Score of 92.33 with 4.2 SD, a mean Oxford Hip Score of 41.44 with 4.53 SD and a mean Moore’s Criteria of Osteo-integration of 3.50 with 0.5 SD (Table 7). Out of the total 60 patients who had having fair outcome, had a mean Harris Hip Score of 74.91 with 5.71 SD, a mean Oxford Hip Score of 29.45 with 4.27 SD and a mean Moore’s Criteria of Osteo-integration of 2.09 with 0.3 SD (Table 7). Out of the total 60 patients, who were having a good outcome, had a mean Harris Hip Score of 84.23 with 3.91 SD, a mean Oxford Hip Score of 33.31 with 5.20 SD and a mean Moore’s Criteria of Osteo-integration of 3.00 with 0.57 SD. That means there is a statistically significant association between the subjective outcome of the patients with scoring (Table 7).

**Table 7:** Comparison of Subjective Outcome with respect to scoring, with standard deviation in parenthesis; p-value deduced via one-way ANOVA test, followed by post-hoc analysis (e.g., Tukey’s test) One-way ANOVA was used for comparisons. SD: Standard deviation. F-statistic degrees of freedom are (between-groups, within-groups) *Indicates statistical significance at p < 0.05. Post-hoc Tukey tests revealed all pairwise comparisons were significant (p < 0.05) for all three measures.* Ref: [12-14]

Subjective outcome	Number of patients	Harris Hip Score, Mean (SD)	Oxford Hip Score, Mean (SD)	Moore's criteria, Mean (SD)
Excellent	36	92.33 (4.20)	41.44 (4.53)	3.50 (0.50)
Good	13	84.23 (3.91)	33.31 (5.20)	3.00 (0.57)
Fair	11	74.91 (5.71)	29.45 (4.27)	2.09 (0.30)
Test Statistic		F(2, 57) = 78.45	F(2, 57) = 35.62	F(2, 57) = 42.18
p-value		<0.001*	<0.001*	<0.001*

We then compared the participants with their Harris hip scores, Oxford hip scores and Moore’s criteria with their age group. It was also observed that out of the total 60 patients, those who were < 40 years of age had a mean Harris Hip score of 92, a mean Oxford Hip score of 34 and a mean Moore’s Criteria of Osteointegration (Table 8). Similarly, those who were 41-50 years of age had a Mean Harris Hip score of 88.75 with SD 11.08, a mean Oxford Hip score of 37.25 with SD 11.02 and a mean Moore’s Criteria of Osteointegration of 3.75 with SD 0.50. Patients who were 51-60 years of age had a mean Harris Hip score of 86.71 with SD 9.25, a mean Oxford Hip Score of 37.57 with SD 7.93 and a mean Moore’s Criteria of Osteo-Integration of 3.29 with SD 0.75. Patients who were 61-70 years of age had a mean Harris Hip Score of 88.47 with SD 8.40, a mean Oxford Hip Score of 38.56 with SD 5.95 and a mean Moore’s Criteria of Osteo-Integration of 3.28 with SD 0.63. 47 Patients who were 71-80 years of age had a mean Harris Hip Score of 84.67 with SD 5.74, a mean Oxford Hip Score of 36.17 with SD 6.86 and a mean Moore’s Criteria of Osteo-Integration of 2.58 with SD 0.66. Patients who were >80 years of age had a mean Harris Hip Score of 85.50 with SD 9.11, a mean Oxford Hip Score of 33.75 with SD 9.03 and a mean Moore’s criteria of Osteo-Integration of 2.75 with SD 0.95. 

There is a statistically significant difference between the age of the patients and Moore’s criteria of Osteo-Integration scoring, but a significant association was not seen between the age of patients and Harris Hip Scoring and Oxford Hip Scoring (Table 8).

**Table 8:** Association between age and clinical and radiological outcome scoring for (N=60) One-way ANOVA was used for comparisons. *Indicates statistical significance at p < 0.05. SD = Standard deviation. F-statistic degrees of freedom are (between-groups, within-groups) For Moore's Criteria, post-hoc analysis showed significant differences between younger (≤70 years) and older (>70 years) age groups. For the ≤40 group, standard deviation cannot be calculated with n=1 and is thus omitted.* Ref: [12-14]

AGE GROUP (YEARS)	NUMBER OF PATIENTS	HARRIS HIP SCORE (MEAN ± SD)	OXFORD HIP SCORE (MEAN ± SD)	MOORE'S CRITERIA (MEAN± SD)
≤40	1	92.00 (NA)	34.00 (NA)	3.00 (NA)
41–50	4	88.75 ± 11.08	37.25 ± 11.02	3.75 ± 0.50
51–60	7	86.71 ± 9.25	37.57 ± 7.93	3.29 ± 0.75
61–70	32	88.47 ± 8.40	38.56 ± 5.95	3.28 ± 0.63
71–80	12	84.67 ± 5.74	36.17 ± 6.86	2.58 ± 0.66
>80	4	85.50 ± 9.11	33.75 ± 9.03	2.75 ± 0.95
Test Statistic		F(5, 54) = 0.56	F(5, 54) = 0.58	F(5, 54) = 3.04
P-value		0.731	0.716	0.017*

## Discussion

The study was conducted as a retrospective study in 60 patients undergoing follow-up for dual mobility total hip arthroplasty in the orthopaedics outpatient department. Around half of our enrolled population belonged to the age group of 61-70 years, with the mean age being 65.77 ± 10.13 years, and the age range extending from 35 to 88 years, and an almost equal distribution of males and females (47% and 53% respectively). The patients were enrolled around 6.15 ± 0.77 years post-operatively, with most of the patients (around 41%) at 6 years post-op.

Postoperative aseptic loosening of the hip joint was noted in only one patient. Our observation of aseptic loosening is quite similar to previous publications in the literature. A meta-analysis by Darrith et al. has reported an incidence of aseptic loosening of the hip joint to be 1.3% in the case of primary dual mobility total hip arthroplasties and 1.4% in the case of revision dual mobility total hip arthroplasties [9]. The authors have also reported 1 case of aseptic loosening of the hip out of 554 hips undergoing dual mobility total hip arthroplasty for treatment of fractures of the femoral neck. Multiple studies, the results of which were pooled by Darrith et al. in their systematic review and meta-analysis, have reported the incidence of aseptic loosening of the hip to be zero or one case out of the total number of cases they have evaluated, similar to the result obtained in our study [9].

Clinical outcomes

We evaluated the post-operative clinical condition of the patients using the Harris’ Hip score and Oxford Hip score. The Harris Hip Score (HHS) is one of the most widely utilised clinician-based outcome measures for assessing hip function, particularly following hip surgery, but also in evaluating various other hip pathologies in the adult population. The HHS evaluates four principal domains: pain (most heavily weighted), physical function, range of motion, and absence of deformity. Söderman and Malchau assessed the inter-rater agreement of the HHS in 58 patients who had undergone THA between 2 and 10 years prior. Independent evaluations by an orthopaedic surgeon and an experienced physiotherapist demonstrated that the HHS possesses high validity and reliability [15]. Similarly, excellent inter-observer reliability has been reported among physical therapists evaluating patients with coxarthrosis scheduled for THA, with kappa values ranging from 0.77 to 0.95 [16]. In our study, we observed a mean Harris Hip Score of 87.38 ± 4.41. These results are consistent with findings by Cantón et al., who reported a mean HHS of 81 ± 22 in a cohort of 31 dual mobility THA implants performed in 30 patients with femoral neck fractures [17]. However, the duration of postoperative follow-up in that study was not specified. Fessy et al. conducted a retrospective multicenter study involving 516 patients who underwent uncemented dual mobility THA. They reported a significant improvement in HHS, from a preoperative mean of 49.6 ± 15.5 to 85.2 ± 14.5 at a median follow-up of 8.7 years [18]. Additionally, they found that larger acetabular cup diameters were associated with improved HHS outcomes [18]. Puch et al. conducted a prospective study of patients receiving second-generation GIROS cementless dual mobility cups. Their cohort included 119 patients younger than 55 years and 444 patients older than 55 years [19]. After a mean follow-up of 11 years (range: 8-15 years), they reported significant improvements in HHS from baseline, with no statistically significant difference between the two age groups [19].

The Oxford Hip Score (OHS) is a 12-question patient-reported outcome measure used to assess pain and function in people with hip problems, especially before and after hip replacement surgery. Each question is scored 0-4, giving a total score between 0 (severe disability) and 48 (no hip problems), with higher scores indicating better hip function.

We obtained the mean Oxford hip score in our study as 37.35 ± 4.60. Lebeau et al. reported a retrospective single-centre case series consisting of 62 patients, with a minimum of 5 years' follow-up, who received a dual mobility acetabular cup with metal reinforcement in revision total hip arthroplasty; to have a Harris Hip Score of 73 and an Oxford hip score of 23.9 [20]. These values were noted to be slightly different from our study, which could be attributed to the difference in patient population.

Griffin et al. investigated the Oxford Hip Score (OHS) as one of the secondary outcome measures in patients undergoing THA for displaced intracapsular hip fractures over a 12-month period. However, the study was limited by a small sample size, enrolling only 20 patients within that timeframe, and was therefore unable to conduct a meaningful analysis of OHS within the study population [21].

In a retrospective, multicentre analysis of 516 patients (541 hips) who underwent uncemented dual mobility THA, Fessy et al. reported a mean postoperative Oxford Hip Score of 19.2 ± 7.6 at a median follow-up of 8.7 years [18]. Similarly, Cantón et al. reported an average Oxford Hip score of 37 (range: 19-48) in a cohort of 30 patients (31 implants) treated with dual mobility THA for femoral neck fractures [16]. However, the specific timing of the postoperative follow-up at which this score was assessed was not provided. In the aforementioned study by Puch et al., the authors also reported significant postoperative improvements in Harris Hip Scores after a mean follow-up of 11 years (range: 8-15 years) [19].

Radiological outcomes

We evaluated the radiological findings in our study by Moore’s criteria for osteointegration. We obtained the overall mean score for Moore’s criteria for osteointegration as 3.10 ± 0.47. The Moore criteria are five radiographic signs used to assess osseointegration of hip prosthesis: absence of radiolucent lines, superolateral buttress, medial stress-shielding, radial trabeculae, and inferomedial buttress. If three or more of these are present, the implant is considered radiographically osteo-integrated [14].

These criteria were originally evaluated by Moore et al. for their ability to predict acetabular osseointegration by reviewing the post-primary and pre-revision radiographs from a series of 119 total hip arthroplasties that underwent revision surgery [14]. We observed that each sign had a high positive predictive value for the presence of bone ingrowth (range, 92.2-96.3%). 97% of the cups with 3 - 5 signs were bone ingrown, whereas 83% of the cups with one or no signs were unstable. When three or more signs were present, the positive predictive value of the radiographic test was 96.9%, the sensitivity was 89.6%, and the specificity was 76.9%. The most sensitive sign for bone growth is often indicated by the absence of radiolucent lines, the presence of medial stress-shielding and the presence of superolateral buttresses. Hence, the authors concluded that the five signs of acetabular osseointegration reliably predicted osseointegration, especially when used in combination [14].

In the previously mentioned study by Canton et al., it was observed on radiographic evaluation that four of Moore’s criteria were present in two cases and three of the criteria in eight other cases [14,16]. In 15 cases, fewer than three criteria were noted. However, none of the cases showed all five criteria. On the basis of these findings, the authors concluded that, in 10 cases out of 25 (40%), full osteointegration of the cup according to Moore’s criteria can be considered [17].

The five criteria combined by Moore have been evaluated in other studies individually by other authors. Manley et al. reported that by the second postoperative year, an increase in bone density in DeLee and Charnley Zone I was observed significantly more often with porous-coated acetabular components than with hydroxyapatite-coated press-fit or threaded components [22].

A finite element study by Levenston et. al.showed that a bone-ingrown cup exerts tensile forces at the inferior region of the acetabulum, producing bone hypertrophy at that region [23]. Jacob et al. experimentally determined that implantation of a device stiffer than the subchondral plate resulted in redistribution of forces to the periphery of the ilium and away from the central cancellous bone [24].

Assessment of subjective outcomes

We assessed the general conditions of the participants at 5-8 years post-operatively by asking for their subjective opinion on their general condition and classifying it as excellent, good or fair. We observed that 60% (n = 36) of the participants reported their condition to be excellent, 22% (n = 13) reported to be good and 18% reported to be fair (n = 11) (Table 6).

Objective outcomes, such as radiological outcomes, were measured by Moore's criteria and Clinical outcomes were measured by the Harris Hip Score and Oxford Hip Score. We then compared the objective and subjective clinical outcomes. We observed that patients who reported excellent outcomes had a greater mean Harris’ hip score (92.33 ± 4.20) as compared to good (84.23 ± 3.91) and fair (74.91 ± 5.71); and this difference was statistically significant. This indicates that patients who had better Harris’ Hip scores were more likely to subjectively feel their post-operative condition to be better.

We also observed that patients who reported excellent outcomes had a greater mean Oxford hip score (41.44 ± 4.53) as compared to good (33.31 ± 5.20) and fair (29.45 ± 4.27); and this difference was also statistically significant. This indicates that patients who had better Oxford Hip scores were also more likely to subjectively feel their post-operative condition to be better.

We also obtained similar results with Moore’s criteria for osteointegration. We observed that patients who reported excellent outcomes had a greater mean score on Moore’s criteria for osteointegration (3.50 ± 0.50) as compared to good (3.00 ± 0.57) and fair (2.09 ± 0.30) outcomes; and this difference was also statistically significant. This indicates that patients who had greater scores on Moore’s criteria for osteointegration were more likely to subjectively feel their post-operative condition to be better.

Similar functional outcomes with Dual mobility THA were also reported by Canton et. al., for patients who underwent the procedure for neck of femur fractures [16]. Our study agrees with these findings by Canton et. al.

Incidence of post-operative complications

We observed only four complications in 60 patients (6.7%) at 5-8 years post-operatively (Table 5). These complications included persistent thigh pain (n = 2), heterotrophic calcification (n = 1) and revision total hip replacement at 6 years (n = 1) (Table 5). Our findings are consistent with those reported by Cantón et al., who also observed a low incidence of postoperative complications in their study involving 31 cases. Reported complications included one Vancouver-type AG periprosthetic fracture, one case of superficial infection, and one instance of persistent thigh pain, accounting for a total complication rate of 9.67% (n = 3) [17]. Notably, the study reported no occurrences of hip dislocation or intra-prosthetic dislocation.

In line with these observations, You et al. conducted a systematic review encompassing 23 studies with a combined total of 7,189 patients. They found a similarly low complication profile associated with dual mobility total hip arthroplasty (DM-THA). Specifically, the incidence of large articulation-related complications was 1.5% (n = 105), while intra-prosthetic dislocation was exceedingly rare at 0.04% (n = 3) [25]. Importantly, their analysis also demonstrated that performing DM-THA for femoral neck fractures did not result in a higher overall complication rate when compared to conventional THA procedures.

Age and outcomes

We compared the age of the participants and the clinical and radiological outcomes. We did not observe any statistically significant association between age and the Harris hip score (p = 0.771) and Oxford hip scores (p = 0.757), indicating that the clinical outcomes were similar across all age groups (Table 8). However, we did observe a statistically significant difference between age and Moore’s criteria for osteointegration (p = 0.020), indicating that the radiological outcomes worsened with advancing age (Table 8).

Limitations

With this study being a retrospective analysis, there is an inherent risk of study selection bias. The study was noted to have a modest study population of 60 patients, which can increase the chance of type II errors and may underrepresent rare complications. The relatively small patient group, as well as the minimum five-year follow-up period, may introduce attrition bias as well. During patient selection, the study included patients who underwent THA “for various purposes,” without stratification by indication. This heterogeneity limits interpretation. While “mid-term” results are informative, the long-term performance (>10 years) of the dual mobility (DM) component, such as polyethylene wear, intra-prosthetic dislocation, and late loosening, remains unknown and would require further studies.

## Conclusions

Our study demonstrates that dual mobility THA provides safe, effective, and durable midterm outcomes across a diverse patient population. Over the follow-up period, we noted that patients exhibited excellent clinical results, which were reflected by marked improvements in pain reduction and functional mobility. Radiological evaluation showed satisfactory implant osteointegration in most cases. Postoperative complications were uncommon, with only a very small proportion of patients experiencing issues such as aseptic loosening, underscoring the low risk profile associated with this implant design. Furthermore, subjective patient satisfaction was high, and clear associations were observed between clinical findings, radiological measures, and patient-reported outcomes.

These findings highlight dual mobility THA as a valuable option in modern hip arthroplasty, particularly for patients at increased risk of instability or dislocation. The strong correlation between radiological and clinical success and patient satisfaction reinforces the implant’s reliability for long-term function and stability. Notably, whilst the radiological outcomes declined slightly with advancing age, clinical improvements remained consistent across all age groups, supporting the use of dual mobility implants even in older populations. Given the low incidence of dislocation, aseptic loosening, and other complications, surgeons may consider dual mobility THA for both primary and revision procedures. Overall, the results support its continued use in clinical practice as a means of achieving stable fixation, sustained functional improvement, and high patient satisfaction over the midterm period.
